# In-vitro studies of phytochemical, Antioxidant, Antibacterial and Enzymatic inhibition of *Oxalis corniculata* whole plant from South Waziristan Tribal District Pakistan

**DOI:** 10.1371/journal.pone.0335026

**Published:** 2026-05-14

**Authors:** Inayat Ullah, Noor Shad Bibi, Abdul Sadiq, Bakhtyar Niazy, Atiq Mohammad Hesam

**Affiliations:** 1 Department of Biochemistry, Abdul Wali Khan University Mardan, Mardan, Khyber-Pakhtunkhwa, Pakistan; 2 Department of Pharmacy, Faculty of Biological Sciences, University of Malakand, Chakdara, Dir (L), Khymber Phwa, Pakistan; 3 Department of Chemistry, Education Faculty, Laghman University, Mehterlam City, Laghman, Afghanistan; Inonu University, Faculty of Pharmacy, TÜRKIYE

## Abstract

**Purpose:**

To analyze phytochemical composition, antioxidant potential, antimicrobial activity and α-glucosidase, cholinesterase and Monoamine oxidase inhibitory potential of *Oxalis corniculata* extract.

**Methods:**

Phytochemicals of the 70% ethanolic extract of *O. corniculata* were quantitatively determined by standard spectrophotometric method. The anti-oxidative and antibacterial potential were investigated by DPPH scavenging test and by well-diffusion method respectively. The p-NPG technique was applied to evaluate α-glucosidase inhibition potential. Moreover, cholinesterase and Monoamine oxidase -A & -B activities were examined by Elman’s method and fluorometric kynuramine deamination test respectively.

**Results:**

Phytochemical study revealed the presence of tannin quantity in range 586 ± 0.05 µg/mg followed by flavonoid; 154.83 ± 0.04 µg/mg and phenolic contents; 135.29 ± 0.01 µg/mg. DPPH radical scavenging of the extract indicates good IC_50_ value 40.74 µg/ml according to the standard ascorbic acid; 25.79 µg/ml. The bacterial inhibition revealed that the extract were high inhibitory against *E. coli, H. influenza* and *k. pneumonia* while showing no inhibition against *Acetobactor aceti*. Similarly, Acetylcholinesterase and Butyrylcholinesterase inhibition result revealed that *O. corniculata* have effective anti-cholinesterase potential with the IC_50_ value of Acetylcholinesterase; 34.54 µg/ml and Butyrylcholinesterase; 8.91 µg/ml. Moreover, *O. corniculata* has good potential against MAO-B inhibition with an IC_50_ value; 60.68 µg/ml, while showing IC_50_ value; 274.23 µg/ml against MAO-A. Furthermore, the inhibitory potential against α- glucosidase of the extract shows significant IC_50_ value (40.59 µg/ml).

**Conclusion:**

*O. corniculata* exhibits potential as a valuable reservoir of natural bioactive compounds endowed with a wide array of health-promoting properties.

## Introduction

It has been a tradition since the dawn of human civilization to treat various illnesses with plants or their derivatives [1,2]. In chemical science, pharmacology, and clinical treatments, medicinal plants are receiving greater interest due to the presence of a variety of bioactive secondary metabolites [3]. Human civilizations revere and protect a wide variety of plants with medicinal, spiritual, and religious value due to their significance to life [4]. Over the years, there have been attempts to investigate the use of plants to cure a variety of diseases including diabetes, neurological disorders like Parkinson’s and Alzheimer’s diseases, and psychiatric ailments like depression [5,6]. Enzymes acting as biocatalysts and are crucial to biological reactions by enhancing the chemical process in the body [7]. Despite the fact that enzymes are necessary for many life processes, some enzymes have been shown to cause illnesses that put life in danger. An excessive intake and the uptake of sugar and lipid by the body is the cause of persistent metabolic disorders including diabetes and obesity [8]. The enzyme amylase is used for the breakdown of complex sugar into simple one’s that are absorbed by the body tissues [9]. A neurotransmitter’s activity is slowed down by the excessive stimulation of acetylcholinesterase and butyrylcholinesterase, which is linked to neurological conditions like Alzheimer’s disease. In certain areas, acetylcholine as acetylcholinesterase is decline quickly by up to 85%, while butyrylcholinesterase levels rise to 165% as the disease progresses [10]. Because of the negative consequences of synthetic medications, plants are receiving more attention as the number of incidences with metabolic ailments and neurological issues increases [11].

*Oxalis corniculata* (Oxalidaceae) is more commonly known by its common names procumbent yellow sorrel, creeping wood sorrel, creeping oxalis. This plant’s leaves are edible, and they have a tart taste similar to lemon. It can be found in America’s tropical areas. It can be found in Pakistan, India, Afghanistan, China, Taiwan, Indonesia, and Japan. *O. corniculata* leaves contain bioactive chemicals like isovitexine, flavonoids, and vitexine-2-O-beta-D-glucopyrunoside. It is a rich source of essential fatty acids like palmitic acid, oleic, linolenic and stearic acids. It possesses various important pharmacological activities like antioxidant, anti-cancer, anthelmintic, anti-inflammatory, antimicrobial, astringent, diuretic, febrifuge, cardio-relaxant and stomachic potential [12].This plant’s juice is used as a digestive aid, an antidote to reptile venom, a treatment for diarrhea, and a diuretic. The stem and bark are used to cure malaria, snakebite, and bronchitis [13,14]. *O. corniculata* leaves and stems contain essential fatty acids such as palmitic, oleic, and linoleic acid, as well as tartaric, citric, and malic acids [15,16]. *O. corniculata* is frequently used as an antiseptic, anti-inflammatory, antimicrobial, wound-healing agent, and to treat piles, cancer, anemia, and stomach issues [17]. It’s been used traditionally to treat kidney stones and bacterial infections [18]. Aqueous extract and silver nanoparticles synthesis of *O. corniculata* were investigated by Das et al. for their ability to suppress germs that cause urinary tract infections (UTIs) and urolithiasis. It has been claimed that the aqueous extract from its leaves efficiently inhibited the formation of struvite stones, leading to the disintegration of the stones. Moreover, its biofabricated silver nanoparticles enhanced the inhibitory efficacy much more [19]. Research published in 2023, discovered that *O. corniculata* holds an assortment of phytochemicals, such as carbohydrates, proteins, phenols, flavonoids, sterols, alkaloids, saponins and tannins [20]. Recently, the non-toxic potential of natural plant substances has received more attention as cancer fighting agents. A study in 2022, reported the ethanolic extract of *O. corniculata* has an apoptotic effect on the MCF-7 breast cancer cell line, suggesting that *O. corniculata* may induce apoptosis through oxidative stress in cancer cells [21]. *O. corniculata* exhibits potential as a valuable reservoir of natural bioactive compounds endowed with a wide array of health-promoting properties.Keeping in view their applications in medicine, this study assessed the phytochemical, antioxidant, antibacterial, and inhibitory properties of *O. corniculata* leaves against key health-related enzymes, including α-glucosidase, acetyl cholinesterase and butyryl cholinesterase, and Monoamine oxidase –A and –B.

## Materials and methods

### Chemical and reagent

All chemical were used of Analytical grade. Ethanol, Methanol, n-hexane, DMSO, NaCO_3_, AlCl_3_, NaCl, NaOH, Folin-Ciocalteu reagent, Gallic acid, Quercetin and 2, 2-Diphenyl-picrylhydrozyl (DPPH), Luria agar (LB-agar), Amoxicillin (GlaxoSmithKline GSK), Phosphate Buffer, p-NPG (p-nitrophenyl-α-D-glucopyranoside), DTNB, Acetylcholine iodide and Butyrylcholine iodide

### Ethical approval and consent to participate

The plant used in the current study is abundantly available and is not endangered species. Plant was collected during summer season after the permission and consultation with Forest Department District South Waziristan Lower, Khyber Pakhtunkhwa, Pakistan.

### Collections and extraction of plants

**Fig 1:**
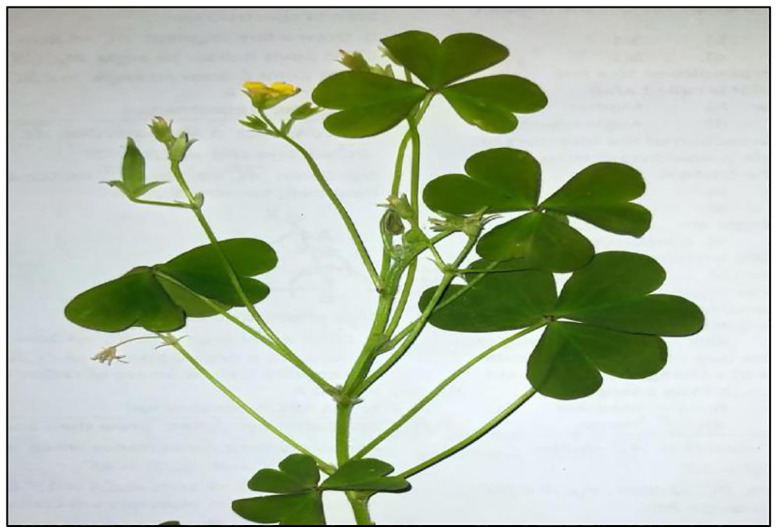
Oxalis corniculata.

The *Oxalis corniculata* plant (Fig 1) was collected from local area of South Waziristan Tribal District, Khyber Pakhtunkhwa, Pakistan. Voucher specimen; AWKUM.Bot.4.1.2 were deposited in the Herbarium at Botany department of Abdul Wali Khan University Mardan, Pakistan for future references. The plant was shaded dried, ground in a mill and extracted by shaking vigorously in 70% Ethanol for 72 hours. Filtered with Whatman- paper and filtrate were concentrated with rotary vacuum evaporated at 55 °C. The concentrated extracts was dried and packed in air tight container at 4 °C for future use.

### Quantitative analysis of different phytochemicals

#### Determination of flavonoid.

Total Flavonoid amount was calculated with the Aluminum chloride calorimetric method by dissolving 0.5 g sample in 5 ml of 80% aqueous ethanol [22]. The extract was left in shaker incubator for 24 hr. The extract was filtered by using Wattmen’s filter paper. 250 µl of filtrate flavonoid sample were mixed with 1.75 ml of distal H_2_O and 75 µl of 5% NaNo_2_ solution. 150 µl of 10% AlCl_3_.6H_2_O was added after 5 min, and left for 6 min. Added 150 µl of 1 M NaOH and 275 µl Distal H_2_O. Mix well the solution by vortex. The blank was performed using distal H_2_O. Quercetin of different concentration (50 mg/ml–0.39 mg/ml) was used as standard. A UV-spectrophotometer was used to measure the absorbance at 215 nm. The sample was performed in triplicates. The total flavonoid in sample were calculated with standard curve calibration of Quercetin in µg/mg of Dry mass (y = 0.0048x + 0.0381. R² = 0.9662).

#### Determination of phenol.

Folin Ciocalteu’s technique was employed to estimate the phenol quantity in the extract. 40 mg crude plant sample were extracted in 1 ml ethanol and kept on Vortex for mixing. Added 1 ml of sample with 1 ml Folin Ciocalteu’s reagent and shacked well. After 5 min, added 1 ml of 20% NaNO_3_ and 1.75 ml distal water. Kept the mixture in dark for 90 min. Gallic acid of different concentration (50 mg/ml-0.39 mg/ml) was used as standard. Absorbance was taken at 750 nm under UV- spectrophotometer. The experiment was carried out in triplicates. The blank was taken using reagent with solvent. In order to plot the calibration curve, standard Gallic acid was used. The total phenol contents in sample were formulated with standard curve calibration of Gallic acid equivalent weight in µg/mg of dry mass {y = 0.0072x + 0.0304. R^2^ = 0.9554} [23,24].

#### Determination of Tannin.

Tannin content was calculated as follows: three replicates of 1 g of sample were extracted with 200 ml of 70% acetone. The extract was left in shaking incubator at 37 °C for 24 hours. Add 250 µl of Folin reagent. After 3 min, 20% of Na_2_CO_3_ were added. A standard stock solution of tannic acid was also made by dissolving 50 mg tannic acid in 100 ml of 70% acetone which then serially diluted (50 mg–0.39 mg). DMSO was used as blank. The solutions were read at 725 nm as described by [25]. The total Tannin contents in sample were formulated with standard curve calibration of Tannic acid equivalent weight in µg/mg of dry mass {y = 0.0004x + 0.1013. R^2^ = 0.9098}.

### Antioxidant assay

The oxidation inhibition potential of the *O. corniculata* was examined by using Brand-Williams and coworker’s procedure [26]. Stock solution of DPPH was prepared with concentration of 24 mg/dl in methanol. In addition, a sample stock solution at a concentration of 1 mg/ml in methanol was prepared. Prepared two fold dilution of different concentration from stock solution of the extract. 2.5 ml of stock DPPH solution were added into 1 ml of each concentration of the extract and then incubated at 27 °C for 30 min. Ascorbic acid was used as positive control. Optical Density was measured at 517 nm by UV-spectrophotometer. The percentage inhibition for radical scavenging activity was calculated by the following formula, and all data were tested in triplicates as mean ± SEM.


$$\%\text{ Inhibition}=\frac{Ac-\;As\;}{Ac}\times \text{ 100}$$

### Anti-microbial assays

#### Bacterial strains.

Four local clinically isolated comprising of three gram negative and one gram positive bacterial strain; *Klebsiella pneumoniae, Acetobactor, Escheria coli* and *Heemophilus* obtained from Drug discovery lab. Department of Biochemistry, Abdul Wali khan university Mardan; Pakistan were used in this study. The stock culture was maintained at 4 °C in LB agar.

### Antibacterial assay

To assess the extract’s antimicrobial properties, the well diffusion method was employed [26]. A 20 ml LB agar was poured into each petriplate, left to solidify for 5 min. 6 mm wells were bored in solidified agar by a sterile cork-borer. Bacterial suspension was inoculated through a sterile stick. 100 μl of plant sample (1 mg/ml) were added into each well through a micropipette and was left to diffuse at room temperature for 1 hour. The plates were wrapped by a tape then placed all the plates in upright position in incubator at 37 °C for 24 hrs. The experiment was performed in triplicate for the extract against each tested organisms. Methanol and distal H_2_O were used as negative control while Antibiotic; *Amoxicillin* (2 mg/ml) was used as positive control. The zone of inhibition against bacterial strain was recorded in millimeter after 24 hours.

### Anti-cholinesterase assays

Elman’s method was followed for the examination of Anti-cholinesterase inhibition potential of the *O. corniculata* L extract using Acetylcholine iodide and Butyrylcholine iodide as substrates [27]. Stock solution of the plant sample was prepared at the concentration of 1 mg/ml. Further prepared two fold concentration (31.25 g/ml–1000 g/ml) from stock solution of the extract. For AchE assay, added 1 ml of the sample solution with 50 µl of AChE (14 mg/ml) and 50 µl of DTNB catalyst in labeled tubes. For BchE assay, also add 1 ml of the sample solution with 50 µl of BChE (14 mg/ml) and 50 µl of DTNB catalyst in labeled tubes. Incubate both assays tubes at 30°C for 15 min. After incubation, added 50 µl of substrate for starting the reaction. Take twice the Optical density through UV-spectrophotometer at 412 nm with time interval for 4 min. All the components of the reaction mixture without plant samples were used as a Blank.

The %inhibition was calculated using the given formulas:


$$\text{V}=\frac{\Delta \text{Abs }(\text{A2}-\text{A1})}{\Delta \text{t }(\text{4}\text{ min})}$$

$$\%\text{ enzyme activity}=\frac{\text{V}}{\text{Control }(\text{V max})\;}\times \text{100}$$

% inhibition=100 − % enzyme activity

Whereas;

V = reaction rate in the presence of inhibitor

V_max_ = reaction rate without inhibitor

### Monoamine oxidase -A and -B inhibition assay

In vitro tests were carried to measure the MAO inhibitory effects of ethanolic extract of *O. corniculata* on human recombinant MAO-A and -B activity. The extract of different concentration (62.5 μg/ml to 1000 μg/ml), and standard MAO inhibitors (selegiline and Safinamide) was examined on human MAO-A and -B enzymes [28]. The MAO-A and -B activity was measured by fluorometric kynuramine deamination assay set up in 96 well plates [29]. 0.1 M potassium phosphate buffer (pH-7.4) was used to carry the enzyme reactions. Prepare substrate solution by diluting the substrate kynuramine to the appropriate concentration for 80 μm MAO-A and 50 μm MAO-B. Add 75 μl of the substrate concentration into each well. Pre-incubate the reaction mixtures with the plant extract and standard inhibitors for 10 minutes at 37 °C to allow the enzyme-inhibitor interactions. After pre-incubation, added 5 μg/ml of MAO-A and 12.5 μg/ml MAO-B enzyme to each well. For proceeding the enzymatic reaction, incubated the plate for 2 0 min at 37 °C. After incubation, add 28 μl of 2N NaOH solution to each well to stop the enzymatic reaction. The deaminated product of kynuramine, which cyclizes spontaneously to 4-hydroxyquinoline was measured fluorometrically using a plate reader at 320 nm excitation and 380 nm emission wavelengths. Calculate the percent inhibition of MAO-A and -B activities for each concentration of the plant extract by comparing the fluorescence intensity with that of the standard.

Calculate the percent inhibition using the following formula;


$$\%\text{ Inhibition}=\{\text{1}-\frac{Absorbanceofsample}{Absorbanceofcontrol}\}\times \text{100}$$

### In-vitro α-glucosidaseinhibition assay

The percent inhibition potential of glucosidase was examined following the procedure described by Mahnashi, M.H., et al., 2022 [30]. Combine 50 μl of each plant extract with two fold dilution (31.05 µg/ml–1000 µg/ml) with 100 μl of glucosidase enzyme (0.5 mg/11 ml). Add 600 μl of phosphate buffer into the reaction mixture. Incubated for 15 minutes at 37 °C thenadded 100 μl of glucopyranoside (15 mg/10 ml), this mixture was kept again incubated for 15 min at 37 °C. The reaction was stop by adding 400 μl of Na_2_CO_3_ to the mixture. The absorbance was measured at 405 nm using double beam spectrophotometer.

The % Inhibitory of the β-glucosidase enzyme was determined using the below formula.


$$\%\text{ inhibition}=\frac{Absorbance\;of\;control-Absorbance\;of\;sample\;}{Absorbance\;of\;control}\;\times \text{1}\text{0}\text{0}$$

### Statistical analysis

The data were analyzed using one-way analysis of variance ANOVA (Dunnett’s multiple comparisons test) using GraphPad prism 8.0 version. The differences between the means of treated and control groups are considered significant at p < 0.05. All tests were carried out in triplicate and the results were presented as means ± standard Error Mean.

## Result and discussion

### Total flavonoid, phenol and tannin content

**Fig 2:**
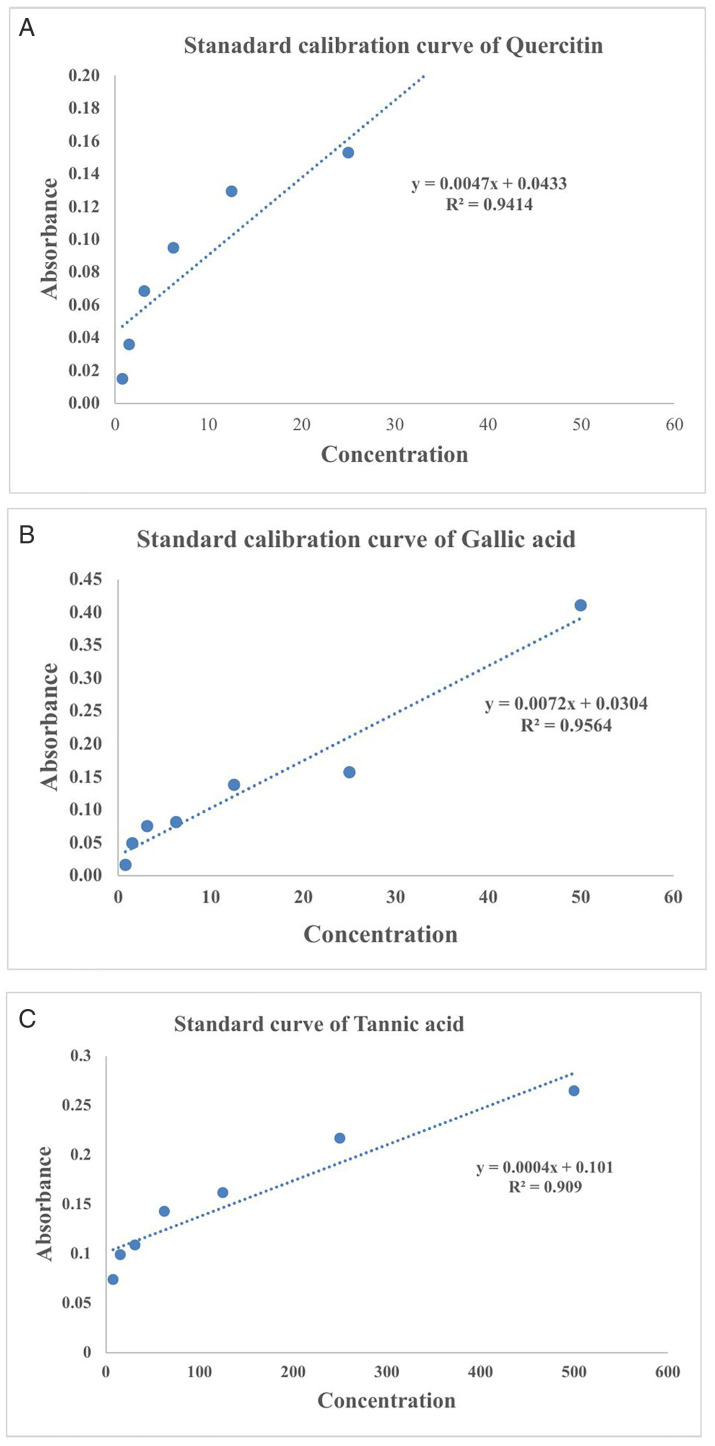
(A). Standard calibration curve of Quercitin. **(B)**. Standard calibration curve of Gallic acid. **(C)**. Standard calibration curve of Tannic acid.

**Fig 3:**
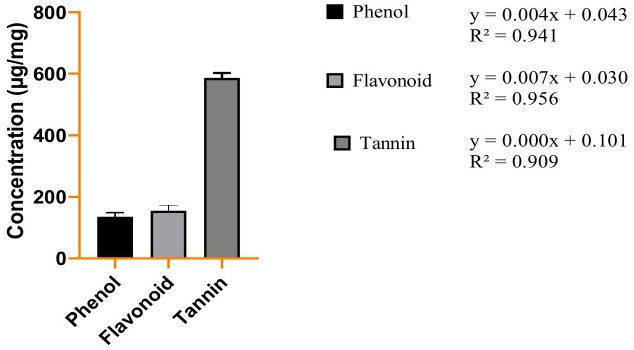
Quantitative analysis of *O. corniculata.*

The last decade has shown increased interest in studying plants as potent sources of alternative drugs to treat several human diseases. It has been also established the importance of phytochemicals for health, and their significance is growing. A diet high in phytonutrients protects against chronic illnesses and a variety of other health issues [31–35]. Previous studies revealed that plant phenolic and flavonoid constituents display significant biological activities [36].The phytochemical contents wereformulated with the calibration curve of the reference drugs Fig 2(A), 2(B), and 2(C). The results showed that the extract have significant amount of phenol, Tannin and Flavonoids contents. This revealed that the amount of Tannin ismaximum in the extract of *O. corniculata*; 586 µg/mg followed by flavonoid; 154.83 µg/mg while phenolic amount are 135.29 µg/mg in range as shown in Fig 3. Our research findings on the quantification of tannin, flavonoid, and phenolic contents in *O. corniculata* provide valuable insights into its phytochemical composition. Comparing these results with previous studies reveals both similarities and differences in the concentrations of these bioactive compounds. A study by Singh et al. (2018) reported a lower tannin content of 320 ± 0.07 µg/mg in *O. corniculata*, suggesting variations in tannin levels due to factors such as geographic location, environmental conditions, and extraction methods [37]. Similar results were reported previously by Muhammad Imran et al., 2020 [38].

### DPPH free radical

In order to ascertain the health advantages and serve as a springboard for more research, the antioxidant potential of *O. corniculata*leaveshas been examined. Previous research also revealed a correlation between the reducing power and the antioxidant features [39]. For this intention, the DPPH scavenging assay is commonly employed for measuring in-vitro antioxidant property of plant extracts as it is quick, reliable and reproducible assay. The obtained results are often expressed as IC_50_ value. Lower IC_50_ value indicates stronger antiradical activity [40]. In this study, DPPH radical removing in 70% ethanol extract of *O. corniculata* indicates the IC_50_ value 40.74 µg/ml. It was determined that the extract showing highest IC_50_ value according to the standard ascorbic acid; 25.79 µg/ml as shown in Table 1. The result are similar to the value of antioxidant of *O. corniculata* (44 µg/ml) reported previously by Sowmya N.M. (2021) [41]. Moreover, [42] reported the value of antioxidant in *O. corniculata* plant (30 µg/ml) which are closely align with our determined value of DPPH radical scavenging [43]. The antioxidant activity of plants is attributed to their rich content of phytochemicals, including phenol compounds, flavonoids, carotenoids, and vitamins. These compounds scavenge free radicals, thereby protecting cells from oxidative damage and reducing the risk of chronic diseases such as cancer, cardiovascular diseases, and neurodegenerative disorders [44].The possible explanation for the antioxidant and anti-inflammatoryproperties include the presence of flavonoids and related polyphenols [45].

**Table 1:** DPPH radical scavenging assay.

Plant Name &Standard	Conc. (µg/ml)	%inhibition ± SEM	IC50 (µg/ml)
Oxalis corniculata	1000 500 250 125 62.5	78.42 ± 0.8 ns 60.46 ± 0.21** 55.09 ± 0.05*** 51.07 ± 0.1*** 48.93 ± 0.1**	40.74
Ascorbic acid	1000 500 250 125 62.5	91.10 ± 0.03*** 84.10 ± 0.04*** 78.66 ± 0.05*** 72.46 ± 0.03*** 65.78 ± 0.02***	25.79

Results are given as mean ± SEM of each triplicates data. Significant statistical differences (Dunnett’s test, p < 0.05) among treatments are expressed in Asterisk. n = 3; ***: significant, ns = not significant.

### Antimicrobial assay

Antibiotics constitute the cornerstone of therapy for bacterial infections. The fact that bacteria are highly genetically variable also means they can quickly escape antibiotics by creating antibacterial resistance. Accordingly, new and better antibiotics are constantly sought for [46]. From the World Health Report on infectious diseases 2000, the most important problem for the WHO into a new millennium would be conquering antibiotic resistance. Therefore, for the management and control of variability in various strains of bacterial species, novel and safer drugs must be provided. Hence, in this present study, antimicrobial activity of the extracts of *O. corniculata leaves* was evaluated by using Disc diffusion method. The result was taken in percent inhibition against gram positive and gram negative bacteria. The result as shown in Table 2 revealed that *O. corniculata* has significant inhibition potential against *H. influenza* and *E. coli* with the % inhibition of 12.33 mm ± 1.20 and 13.67 mm ± 0.88 respectively. While have low zone of inhibition against *K. pneumoniae*; 7.00 mm ± 0.58 and have zero inhibition value against *A. aceti*according to the average zone of inhibition of the reference drug, Ampicillin against each bacterial strain; 7 mm, 11 mm, 8 mm and 13 mm respectively. The findings are consistent with those from a previously reported research of *O. corniculata*.It was previously discovered that the ethanol and methanol extracts’ antibacterial action was due to the presence of phenolic chemicals [47], In aqueous extract, *O. corniculata* exhibited significant antibacterial activity [48], *O. corniculata extract*in 80% ethanol exhibited antibacterial action and *O. corniculata* showed a strong antibacterial action to some extent. The current study demonstrates the plant’s antibacterial properties and proposes that it could be utilized to treat ailments caused by these bacteria in humans.

**Table 2:** Antibacterial activity of *O. corniculata* extract.

	*H. influenza*		*E.coli*		*A. aceti*		*K. pneumoniae*	
Plant name	Mean/mm	±SEM	Mean/mm	±SEM	Mean/mm	±SEM	Mean/mm	±SEM
*O. corniculata*	12.33	1.20	13.67	0.88	0	0	7.00	0.58
Ampicillin	7	0	11	0	11	0	17	0

### Anti-cholinesterase assay

Alzheimer’s disease (AD) is the most serious neurological condition affecting people today. It is typified by a reduction in cognitive and mental abilities and is directly linked to a loss of cortical cholinergic neurotransmission [49]. The primary enzymes that hydrolyze and deactivate acetylcholine, a neurotransmitter, are acetylcholinesterase (AChE) and butyrylcholinesterase (BChE).The synthetic AChE and BChE inhibitors showed relatively modest therapeutic benefits in terms of memory preservation and noticeable side effects. In order to treat and control AD holistically and effectively, natural sources of co-inhibitors of AChE and BChE activities are being used [50].This plant extract gives a significant inhibition of AChE compared to thestandard drug; Galantamine. The effects of AchE inhibition linked to AD were assessed in our study. It was determined that the Acetylcholinesterase and Butyrylcholinesterase inhibition potential of the *O. corniculata* L. extractare much effective with an IC_50_ value of 34.54 µg/ml and 8.906 µg/ml respectively as compared to the IC_50_ value 0.83 µg/ml of the standard drug; Galantamine as shown in the Table 3. Imran et al, 2020, also reported inhibitory potential of cholinesterase in *O. corniculata* extract with the IC50 value of AchE; 49.52 μg/mL and BchE; 29.12 μg/mL [38].These findings regarding the plant extract’s ability to inhibit cholinesterase showed potential in preserving brain function and reducing the risks associated with AD.

**Table 3:** Anti-cholinesterase activity of *O. corniculata.*

		Acetylcholinesterase assay		Butyrylcholinesterase assay	
Plant Name & Standard	Conc. (µg/ml)	% inhibition ± SEM	IC50 (µg/ml)	% inhibition ± SEM	IC50 (µg/ml)
*Oxalis corniculata*	1000 500 250 125 62.5	90.7 ± 0.04 ns 84.74 ± 0.04 ns 81.42 ± 0.03 ns 76.16 ± 0.02 ns 63.71 ± 0.04**	34.54	88.69 ± 0.04** 83.63 ± 0.03*** 81.43 ± 0.03** 79.26 ± 0.04** 76.80 ± 0.04**	8.91
Galantamine	1000 500 250 125 62.5	94.50 ± 1.02*** 91.27 ± 1.13*** 86.73 ± 0.66*** 82.13 ± 0.87*** 77.00 ± 0.4***	0.83	93.12 ± 0.06*** 87.45 ± 0.05*** 83.34 ± 0.05*** 78.88 ± 0.03*** 72.14 ± 0.07***	2.7

Results are given as mean ± SEM of each triplicates data. Significant statistical differences (Dunnett’s test, p < 0.05) among treatments are expressed in Asterisk. n = 3; ***: significant, ns = not significant.

### Monoamine oxidase inhibition

The effect of the *O. corniculata* L.extract on the MAO enzyme is given in the Table 4. The result showed that *O. corniculata* extract has good potential against MAO-B inhibition with an IC_50_ value; 60.68 µg/ml with compare to the reference drug; safinamide having IC_50_ value 0.12 µg/ml while showing IC_50_ value; 274.23 µg/ml against MAO-A which is not more effective inhibition potential as compared to the standard drug selegiline with IC_50_ value; 2.10 µg/ml. Our investigation has led us to the conclusion that the extract has dose-dependently inhibited MAO-B enzyme activity. The extract did not, however, exhibit any notable MAO-A inhibiting activity. Our antioxidant and anti-cholinesterase research findings are at odds with the results of the MAO-A enzyme inhibition examination. However, the reason behind this is unknown. Some other studies revealed that different doses of *O. corniculata*extract increased significantly memory retention and retrieval. *O. corniculata* L. extract increases memory retention and retrieval which may be due to the presence of antioxidants such as flavonoids, coumarins, tocopherols, and phenolic acids [51].

**Table 4:** Monoamine oxidase –A and –B inhibition potential of *O. corniculata.*

Plant Name& Standard	Conc. (µg/ml)	Monoamine oxidase –A Inhibition		Monoamine oxidase –B Inhibition	
		% MAO-A	IC50 (µg/ml)	% MAO-B	IC50 (µg/m)
*Oxalis corniculata*	1000 500 250 125 62.5	62.42 ± 0.43*** 55.56 ± 1.06*** 48.90 ± 2.45*** 43.40 ± 0.82*** 35.33 ± 1.66***	274.23	79.37 ± 1.04^***^ 72.37 ± 0.54^***^ 65.30 ± 2.61^***^ 58.42 ± 1.05^***^ 50.52 ± 2.52^***^	60.68
Selegiline MAO-A & Safinamide MAO-B	1000 500 250 125 62.5	92.81 ± 0.12*** 87.52 ± 0.30*** 84.72 ± 0.10*** 81.45 ± 0.42*** 77.10 ± 0.32***	2.10	95.85 ± 0.18*** 91.59 ± 0.30*** 87.75 ± 0.14*** 84.47 ± 0.49*** 81.12 ± 0.34***	0.12

Results are given as mean ± SEM of each triplicates data. Significant statistical differences (Dunnett’s test, p < 0.05) among treatments are expressed in Asterisk. n = 3; ***: significant, ns = not significant.

### Alpha-glucosidase inhibition

Recent advances in understanding the action of intestinal enzymes, such as α-amylase and α-glucosidase, have resulted in the development of novel pharmacological medications. These enzymes are critical for the breakdown and absorption of carbohydrates.A high postprandial blood glucose response, which is higher than fasting blood sugar levels, is more strongly associated with the risk of cardiovascular diseases and diabetes complications. Intestinal lumen secretedα-glucosidase enzymes and its function is to convert starch and oligosaccharides to monosaccharides for absorption.It was assumed that limiting the action of such digestive enzymes would delay the decomposition of starch and oligosaccharides, producing a decrease in glucose absorption and, therefore, a reduction in postprandial blood glucose levels rising [52]. Alpha-glucosidase inhibitors prevent carbohydrates from being absorbed and digested quickly. Acarbose and miglitol compete to block α-glucosidase, lowering starch and disaccharide absorption. Thus, one of the therapeutic strategies to utilize to reduce postprandial (PP) blood glucose levels in diabetes patient is to limit carbohydrate absorption after food consumption. Inhibiting α-amylase and α-glucosidase can reduce high postprandial (PP) blood glucose peaks in diabetes [52]. α-amylase inhibitors limit carbohydrate digestion and absorption, functioning as anti-nutrient. Acarbose is a complex oligosaccharide that delays the breakdown of carbohydrates. It stops pancreatic amylase from degrading starch. Synthetic inhibitors cause side effects such as stomach pain, diarrhea, and soft feces in the colon. To address this, there is a need to focus on scientific research into herbal treatments that have less negative effects. Based on the plant’s traditional use as an anti-diabetic and anti-hyperlipidemia agent, the current study was conducted in vitro to support the folklore claim. Our findings show that the ethanolic extract of O. corniculata effectively inhibits the α-glucosidase enzyme in vitro. There was a dose-dependent increase in percentage inhibitory activity against α-glucosidase enzyme by the O. corniculata extract. At a concentration 62.5 µg/ml of extract showed a percentage inhibition 38.93 ± 0.02 and for 1000 µg/ml it was 61.47 ± 0.04 (Table 5). The PHE gave an IC_50_ value; 40.59 µg/ml. The IC50 value of standard drug acarbose was found; 20.25 µg/ml µg/ml (Table 5).

**Table 5:** Alpha – Glucosidase activity of O. corniculata.

Plant Name &Standard	Conc. (µg/ml)	% inhibition ± SEM	IC50 (µg/ml)
*Oxalis corniculata*	1000 500 250 125 62.5	61.47 ± 0.04*** 55.47 ± 0.05*** 50.40 ± 0.02*** 45.20 ± 0.03*** 38.93 ± 0.02***	40.59
Acarbose	1000 500 250 125 62.5	88.04 ± 0.08*** 82.43 ± 0.17*** 77.56 ± 0.14*** 72.08 ± 0.08*** 67.53 ± 0.11***	20.25

Results are given as mean ± SEM of each triplicates data. Significant statistical differences (Dunnett’s test, p < 0.05) among treatments are expressed in Asterisk. n = 3; ***: significant, ns = not significant.

## Conclusion

The present study demonstrates that *Oxalis corniculata* is a rich source of bioactive phytochemicals, with tannin content of 586 ± 0.05 µg/mg, flavonoids 154.83 ± 0.04 µg/mg, and phenolic compounds 135.29 ± 0.01 µg/mg, which contribute to its strong antioxidant potential. The extract showed significant DPPH radical scavenging activity with an IC₅₀ of 40.74 µg/ml, compared to standard ascorbic acid (25.79 µg/ml), confirming its effective free-radical quenching ability. The antibacterial evaluation revealed strong inhibitory activity against *E. coli*, *H. influenzae*, and *K. pneumoniae*, while no inhibition was observed against *A. aceti*, indicating selective antibacterial potential. Neuroprotective assays demonstrated potent inhibition of acetyl cholinesterase (IC₅₀ = 34.54 µg/ml) and butyryl cholinesterase (IC₅₀ = 8.91 µg/ml), highlighting its promising anti-cholinesterase capacity. Additionally, the extract showed notable inhibition of MAO-B (IC₅₀ = 60.68 µg/ml) compared to its weaker effect on MAO-A (IC₅₀ = 274.23 µg/ml), suggesting potential in managing Parkinson’s-related oxidative stress. Moreover, significant α-glucosidase inhibition was observed with an IC₅₀ value of 40.59 µg/ml, indicating its potential role in controlling postprandial hyperglycemia. Overall, these findings highlight *O. corniculata* as a potent source of natural antioxidants, antimicrobials, neuroprotective, and antidiabetic agents. Due to the significant amount of tannin, flavonoidand phenolic compounds, the extract shows a good antioxidant, antimicrobial and enzymatic inhibitory potential. *O. corniculata* plant is common edible food plant and is locally approved as plant having traditional values. All the studyresults indicate that since the plant possess potent inhibitory potential against bacteria, oxidation free radical, α – glucosidase and cholinesterase. These findings pave the way for further exploration of this plant as a natural source of antibiotic, antioxidant and metabolic disorders. This study not only highlights the significance of natural products in modern medicine but also emphasizes the importance of multidisciplinary research in unlocking their full potential for human health and well-being. Further investigation is necessary for the isolation and characterization of the secondary metabolites of *O. corniculata* that could yield crucial components which can be employed to the enzymatic inhibition on the human body.

## Abbreviations

DPPH: 2,2-Diphenyl-picrylhydrozyl
p-NPG: p-nitrophenyl-α-D-glucopyranoside
µg: microgram
ml: milliliter
µl: micro liter
μm: micrometer
DMSO: dimethyl sulfoxide
NaCO_3_: sodium carbonate
AlCl_3_: aluminum chloride
NaCl: sodium chloride
NaOH: sodium hydroxide
DTNB: 5,5’-dithiobis-2-nitrobenzoic acid
SEM: standard error mean
LB agar: Luria broth agar
AchE: acetyl cholinesterase
BchE: butyryl cholinesterase
AD: Alzheimer disease
WHO: World Health Organization
